# Folic Acid-Modified Milk Exosomes Delivering c-Kit siRNA Overcome EGFR-TKIs Resistance in Lung Cancer by Suppressing mTOR Signaling and Stemness

**DOI:** 10.7150/ijbs.99954

**Published:** 2025-01-01

**Authors:** Zihan Xu, Li Wang, Li Tu, Tao Liu, Yong Zhang, Yingying He, Guixiu Xiao, Ganlu Ouyang, Xuelei Ma, Feng Luo

**Affiliations:** 1 Department of Medical Oncology, Cancer Center, West China Hospital, Sichuan University, Chengdu, Sichuan, China, 610041.; 2 Lung Cancer Center, West China Hospital, Sichuan University, Chengdu, Sichuan, China, 610041.; 3 Institute for Breast Health Medicine, State Key Laboratory of Biotherapy, West China Hospital, Sichuan University and Collaborative Innovation Center, Chengdu, Sichuan, China, 610041.; 4 Department of Biotherapy, West China Hospital and State Key Laboratory of Biotherapy, Sichuan University, Chengdu, Sichuan, China, 610041.

**Keywords:** non-small cell lung cancer, EGFR-TKIs resistance, c-kit, milk exosomes, stemness

## Abstract

The EGFR-TKIs (epidermal growth factor receptor-tyrosine kinases inhibitors) offer significant benefits to lung cancer patients with sensitive EGFR mutations; however, the development of acquired resistance poses a significant challenge and leads to poor prognosis. Thus, exploring novel therapeutic strategies to overcome EGFR-TKI resistance is urgently needed. This study introduces an innovative approach utilizing folic acid-modified milk exosomes loaded with c-kit siRNA (FA-mExo-siRNA-c-kit) to target EGFR-TKI resistance in lung cancer. Initially, gefitinib-resistant lung cancer cells exhibited stemness characteristics, including an epithelial-to-mesenchymal transition phenotype and elevated ABCG2 expression, which were closely regulated by c-kit. Subsequent treatment with FA-mExo-siRNA-c-kit demonstrated effective suppression of c-kit expression and attenuation of stemness traits *in vitro*, reducing gefitinib resistance. In xenograft and liver metastasis models, sequential administration of FA-mExo-siRNA-c-kit and gefitinib resulted in decreased tumor growth and prolonged survival. Mechanistically, c-kit was found to regulate the AKT/mTOR/4EBP1/eIF4E axis, promoting stemness and gefitinib resistance in lung cancer cells. This study unveils a novel mechanism of EGFR-TKI resistance involving the c-kit/mTOR pathway and proposes a promising therapeutic strategy for EGFR-TKI-resistant lung cancer, particularly with liver metastasis, using FA-mExo-siRNA-c-kit, suggesting potential for improved patient outcomes and warranting further investigation.

## Introduction

The EGFR-TKIs (epidermal growth factor receptor-tyrosine kinases inhibitors) have emerged as an effective therapeutic approach for lung cancer patients with EGFR mutations. However, several evidences suggest the inevitability of acquired resistance to EGFR-TKIs in these individuals 1. Presently, EGFR-TKI resistance in lung cancer arises through various mechanisms, including secondary mutations in EGFR (e.g., T790M), activation of alternative signaling pathways (e.g., MET, HER2), phenotypic transformation (small cell transformation). Various strategies have been developed to address these resistance mechanisms, including the use of next-generation EGFR inhibitors (e.g., Osimertinib), combining EGFR-TKIs with inhibitors of alternative pathways or traditional therapies like chemotherapy or radiation, and targeting downstream signaling molecules 2, 3. However, approximately 20-30% of patients with acquired resistance still have unclear underlying causes and lack effective measures to overcome resistance. Given the liver's frequent involvement as a metastatic site in lung cancer, the emergence of liver metastasis following EGFR-TKIs resistance is prevalent 4. This occurrence of post-resistance liver metastasis poses a significant challenge for the following treatment including chemotherapy, targeted therapy and immunotherapy, thereby impacting overall survival of these patients 5, 6. Therefore, there is an urgent need to explore novel molecular mechanisms of EGFR-TKI resistance and develop innovative therapeutic approaches, particularly for individuals with liver metastases.

Stemness phenotype transformation is one of the characteristics of self-remodeling of cancer cells and frequently arises during the development of drug resistance 7-9. Recent studies have unveiled that lung cancer cells resistant to EGFR-TKIs exhibit characteristics indicative of a stemness phenotype. Ahmad A *et al.* 10 demonstrated that erlotinib-resistant lung cancer cells possessed enhanced metastatic potential and displayed elevated expression levels of stemness-associated genes, including *Nanog*, *CD133*, *Oct4*, and *Sox2*. Moreover, our previous study confirmed that gefitinib-resistant lung cancer cells exhibited enhanced self-renewal capacity and resistance to chemotherapy 11. Consequently, the identification of key genes associated with stemness-phenotype in EGFR-TKIs-resistant lung cancer cells holds promise for overcoming resistance and improving outcomes for these patients.

c-kit, a pivotal member of the tyrosine kinase family, engages in diverse downstream signaling cascades upon binding with its ligand stem cell factor (SCF). These downstream pathways include RAS/ERK, PI3-kinase, SRC, JAK/STAT, WNT, and NOTCH, collectively modulating cell differentiation, proliferation, and metabolism 12-18. In our previous study, an upregulation of c-kit expression was observed in gefitinib-resistant lung cancer cells; it was intricately linked to the regulation of the stemness phenotype in these cells 11. Therefore, targeting the expression of c-kit in these cells might hold a promise for overcoming gefitinib resistance.

Currently, exosomes, the natural nanoscale extracellular vesicles, have gained widespread attention in drug delivery due to their notable advantages, including stability, biocompatibility, permeability across the blood-brain barrier, low immunogenicity, and toxicity 19. In particular, milk-derived exosomes offer increased yield and accessibility compared to exosomes derived from other sources. These exosomes hold promise as drug delivery carriers, with preclinical research demonstrating their anti-tumor effects 20. Folic acid (FA) is widely recognized for its high affinity to folate receptor alpha (FR-α), which makes it a popular choice for modifying drug carriers to achieve targeted delivery 21, 22. FR-α is a glycosylphosphatidylinositol-anchored receptor that is overexpressed in various cancer types, including ovarian, breast, and lung cancers, providing a selective target for FA-modified cancer therapies 23-25. Compared to other targeting receptors, such as transferrin receptors or integrins, FA/FR-α offers higher specificity, lower immunogenicity, and better compatibility with targeted delivery systems 26, 27. In this study, we found that FR-α is significantly overexpressed in gefitinib-resistant lung cancer cells, indicating that FA modification can enhance the targeting ability of milk exosomes towards these cells, thereby reducing off-target effects.

The current study aimed to explore the anti-tumor effects of FA-modified milk-derived exosomes loaded with c-kit siRNA on gefitinib-resistant lung cancer, both *in vitro* and *in vivo.* Moreover, the current study also attempted to elucidate the molecular mechanism underlying gefitinib resistance, specifically focusing on the involvement of the c-kit signaling pathway in driving the stemness phenotype transformation.

## Materials and Methods

### Cell lines

The human NSCLC (non-small cell lung cancer) cell lines PC9 and HCC827 were purchased from the Cell Bank of the Chinese Academy of Sciences (Shanghai, PR China). These cell lines were cultured in RPMI-1640 medium (Gibco) supplemented with 10% fetal bovine serum (FBS, Gibco) and incubated in a humidified incubator at 37 °C with 5% CO_2_. The EGFR-TKIs-resistant NSCLC cell lines PC9/Gr and HCC827/Gr were generously provided by Professor Feng Luo from the Lung Cancer Center, Laboratory of Lung Cancer, West China Hospital of Sichuan University. The resistant cell line was established as previously described 28.

### Reagents and chemicals

Gefitinib, Rapamycin, and MK2206 were purchased from MCE MedChemExpress. All reagents were prepared and stored according to the provided instructions. The primary antibodies against AKT (#9272), p-AKT S473 (#9271), mTOR (#2972), p-mTOR S2448 (#2971), 4EBP1 (#9452), p-4EBP1 S65 (#9451), eIF4E (#9742), and p-eIF4E Thr70 (#9455) were obtained from Cell Signaling Technology, while those against Kit (ab32363), ABCG2 (ab207732), OCT4 (ab200834), ALDH1A1 (ab134188), Nanog (ab203919), PROM1 (ab222782), N-cadherin (ab76011), E-cadherin (ab231303), Vimentin (ab20346), and SNAIL (ab216347) were obtained from Abcam. Moreover, a Folate receptor-a (SAB4500989) and β-actin (R23613) were purchased from Merck and ZenBioScience, respectively. The secondary antibodies, including HRP (Horseradish Peroxidase)-conjugated goat anti-rabbit (A0208) and anti-mouse (A0216), were sourced from Beyotime Biotechnology.

### Animals

Six-week-old female mice [Athymic nude (BALB/c) mice and wild-type (C57BL/6J WT) mice] were purchased from Beijing Huafukang Biotechnology Company (Beijing, China). All the animal experiments were conducted following the Guidelines for the Care and Use of Laboratory Animals as outlined by the National Academy of Sciences and published by the National Institute of Health. The experimental protocols were approved by the Experimental Animal Ethics Committee of West China Hospital, Sichuan University (Approval No. 20230510006).

### Exosomes isolation

Exosomes were isolated from raw milk using a differential centrifugation method, following established protocols 29. Initially, the milk was preheated in a warm water bath at 37 °C for 10 min. Subsequently, acetic acid was added at a ratio of 100:1 (milk: acetic acid) and allowed to stand at room temperature for 5 min to ensure complete denaturation of proteins. After denaturation, the mixture was centrifuged at 4,500×g for 30 min at 4 °C. The resulting supernatant was then filtered through a 0.22-μm filter and centrifuged again at 8000×g for 30 min at 4 °C. The supernatant obtained from this step was carefully collected and transferred to a 38-mL ultracentrifuge tube. Subsequently, the supernatant was centrifuged at 120,000×g for 75 min at 4 °C using an ultracentrifuge (Beckman Coulter, Brea, California) equipped with a 45SW Ti horizontal angle rotor to obtain an exosomal pellet. The exosome pellet was washed twice with PBS (phosphate buffered saline) and then resuspended in PBS. The suspension of exosomes was aliquoted and stored at -80 °C at a concentration of 1 mg exosomal proteins/mL until further use.

### Electron microscopy

The exosome samples were diluted with PBS to achieve at least 3 concentration gradients. Carbon-coated grids were rendered hydrophilic by allowing the diluted sample to adsorb for 10 min. Then, the excess liquid was removed using filter paper. Subsequently, the grids were stained with 1% uranyl acetate for 30 s. After removing excess uranyl acetate with filter paper, the grids were examined, and images were captured using a transmission electron microscope (Hitachi, Japan).

### Nanoparticle tracking analysis

For analysis, the exosome samples were diluted in PBS to achieve a concentration within the recommended range (1×10^7^-1×10^9^ particles/mL) and subsequently vortexed for 1 min. Following this, the samples were loaded into the sample chamber at room temperature, and a single 60 s video was captured for each sample. The videos were subjected to analysis using NTA3.2 software, which identified and tracked the center of each particle undergoing Brownian motion to measure the average distance moved by the particles on a frame-by-frame basis. The number and size of the exosomes were directly assessed using the NS300 instrument (Zetasizer Nano ZS; Malvern Instruments, Malvern, UK).

### Preparation of the FA-modified exosomes

The NEPA21 high-efficiency gene transfection system (NEPA GENE, Japan) was used to load exosomes with siRNA using electroporation. Initially, exosomes were diluted to a concentration of 0.5 mg/mL in PBS and then mixed with 80 nM of siRNA targeting c-kit or fluorescence-labeled control siRNA (GenePharma, Shanghai, China). The entire procedure was carried out in ice-cold 0.4-cm cuvettes, and electroporation was conducted at 200 V for a pulse duration of 10-15 ms. Samples were maintained on ice for at least 10 min both before and after electroporation pulse, followed by dilution in PBS and subsequent centrifugation at 120,000×g for 70 min.

FA has been employed to functionalize nanoparticles in order to enhance their efficacy in delivering drugs to tumor sites, as described in previous studies 30. Briefly, exosomes at a concentration of 10 mg/mL were mixed with 100 μL of NaHCO_3_ (Sodium Bicarbonate) buffer (1M, pH 8.4) and 100 μL of freshly prepared FA NHS (N-hydroxysuccinimidyl) ester solution (containing 2.5-3 mg of activated FA dissolved in 0.1 mL of 0.1 N NaOH). The samples were incubated at room temperature for 1 h. After incubation, the samples were diluted with PBS and centrifuged at 120,000×g for 70 min. The exosome pellet obtained after centrifugation was washed twice with PBS and finally resuspended in PBS.

### In vivo biodistribution of exosomes

In order to examine the biodistribution of intravenously administered exosomes, athymic nude (BALB/c) mice (n = 4 per group) were employed. Initially, mExos were labeled with a near-infrared fluorescent dye DiR (20 μM). The sample was incubated at 37°C for 30 min and then centrifuged at 120,000×g for 70 min to remove unbound dye. The labeled exosomes were sterilized by filtration through a 0.22-μm filter. Subsequently, the mice were administered with a single dose of 100 μL DiR-labeled exosomes (60 mg/kg mExo protein).

The distribution of FA-mExo *in vivo* was monitored using the IVIS (*in vivo* imaging system) Spectrum (PerkinElmer, America) at various time intervals (2 h, 4 h, 8 h, 24 h, 48 h, and 72 h). After 24 h of treatment, the animals were euthanized, and different organs were collected for *ex vivo* imaging using the IVIS Spectrum.

### In vivo toxicity studies

Similar protocols were implemented using wild-type (C57BL/6J WT) mice to explore the effects of FA-mExo-siRNA-c-kit (60 mg/kg mExo protein). These mice received daily intravenous injections through the tail vein for 7 consecutive days, followed by blood collection 24 hours after the final administration. Hematological analyses were performed on the collected blood samples using a Cell Dyn 3500 hematology analyzer (Abbott Laboratories, Santa Clara, CA). Serum samples were subjected to various biochemical assessments of liver and kidney function using an automated AU640® Chemistry Analyzer (Beckman Coulter, Inc., Brea, CA, USA), while IgE (Immunoglobulin E) levels were determined using ELISA (Enzyme-linked Immunosorbent Assay). Moreover, vital organs were excised, fixed in 4% paraformaldehyde, and subjected to H&E staining (hematoxylin-eosin staining) for histopathological examination.

### Sanger sequencing

The complete sequences of *EGFR* (NC_000007.14, Gene ID: 1956), *BRAF* (NC_000007.14, Gene ID: 673), and *PIK3CA* (NC_000003.12, Gene ID: 5290) genes were retrieved from the NCBI database. Primers were designed based on the targeted mutation sites using Primer 5 software. PCR (Polymerase Chain Reaction) conditions were optimized according to the calculated Tm (melting temperature) value of the designed primers, and the PCR products were subjected to 1% agarose gel electrophoresis for visualization. Following electrophoresis, the PCR products were recovered, and the purified PCR samples were submitted to Qingke Biotechnology for Sanger sequencing analysis. The obtained sequences were aligned and analyzed against the NCBI database.

### Transcriptome sequencing (RNA-seq)

Total mRNA was extracted using RNeasy Mini Kit (QIAGEN, Germany) followed by RNA-seq analysis conducted at Sinotech Genomics (Shanghai, China). Briefly, 2 μg of RNA from each sample was utilized to construct a cDNA library, which was subsequently sequenced on the Illumina HiSeq 5000. Raw reads were aligned to the NCBI database using Bowtie software. Subsequently, a differential expression analysis was conducted using the R software package “edgeR” (2.6.2), with significant thresholds set at an adjusted *P*-value of <0.05 and FDR (false discovery rate) of <0.01.

### Quantitative real-time PCR (qRT-PCR)

RNA was extracted from cells at 48 hours post-siRNA transfection with TRIzol reagent (Invitrogen). cDNA was produced from reverse transcription using avian myelobastosis virus (AMV) reverse transcriptase (TaKaRa). qRT-PCR was performed with respective primers listed in Supplementary Table 1, cycling conditions as follows: 95 °C for 30s; 95 °C for 5s and 72 °C for 30s, which was repeated for 40 cycles. All experiments were carried out in triplicate.

### Xenografts assay

Female BALB/c nude mice were subcutaneously injected with 100 μL of PC9/Gr cell (1 × 10^6^ cells) suspension. All tumor-bearing mice were randomly divided into five groups (n = 5 per group). The groups were treated with PBS, Gefitinib (5 mg/kg), FA-mExo-NC + Gefitinib (60 μg/kg, 5 mg/kg), FA-mExo-c-kit (60 μg/kg), and FA-mExo-c-kit + Gefitinib (60 μg/kg, 5 mg/kg) using intratumor injection (mExo) or gavage (Gefitinib) starting 10 days after tumor inoculation. The treatment regime was administered daily for 14 days. The tumor volume was measured every other day following the initiation of treatment. Meanwhile, the diet, weight, and survival status of mice were closely monitored throughout the study. At 16 days of initial dosage, all mice were euthanized by dislocating the cervical vertebra. Tumor tissue and vital organs were collected, fixed in 4% paraformaldehyde, and immediately frozen in liquid nitrogen for further analysis.

### Metastatic liver tumor animal model

Subcutaneous xenografts of drug-resistant lung cancer (PC9/Gr cells) were established using 6-8 weeks-old female BALB/c nude mice following the previously described protocol. Once the tumor volume reached approximately 500-750 mm^3^, the mice were euthanized, and the tumors were carefully dissected and cut into uniform pieces, which were then stored in pre-chilled PBS for subsequent use. The surgical procedure for establishing liver metastases was conducted as previously outlined 31.

After confirmation of the successful establishment of metastatic liver tumor models, the mice were randomly divided into five groups (n = 5 per group), including PBS, gefitinib, FA-mExo, FA-mExo-siRNA-NC+gefitinib, and FA-mExosiRNA-c-kit+gefitinib groups. The administration regimen included intravenous injection of mExo in tail (60 μg/kg) every other day for 12 days, and gefitinib administration via gavage at a dose of 5 mg/kg for 12 days. Throughout the treatment period, close monitoring of the mice's diet, weight, and survival status was carried out. Liver metastases in mice were detected using IVIS Spectrum during the treatment course. On the 14^th^ day of the initial dosage, all mice were euthanized by dislocating the cervical vertebra. The contents of TNF-α, IFN-γ and IL-12 were determined by ELISA. Tumor tissue and vital organs were collected and fixed in 4% paraformaldehyde. Subsequently, the specimens were immediately frozen in liquid nitrogen for further analysis.

### Statistical analysis

All the data were expressed as mean ± SEM (Standard error of measurement). The comparisons between two groups were conducted using a *t*-test, while comparisons among more than two groups were performed using one-way ANOVA (Analysis of Variance). A *P*-value of <0.05 was considered statistically significant.

## Results

### The characteristics of gefitinib-resistant lung cancer cells

**Figure 1:**
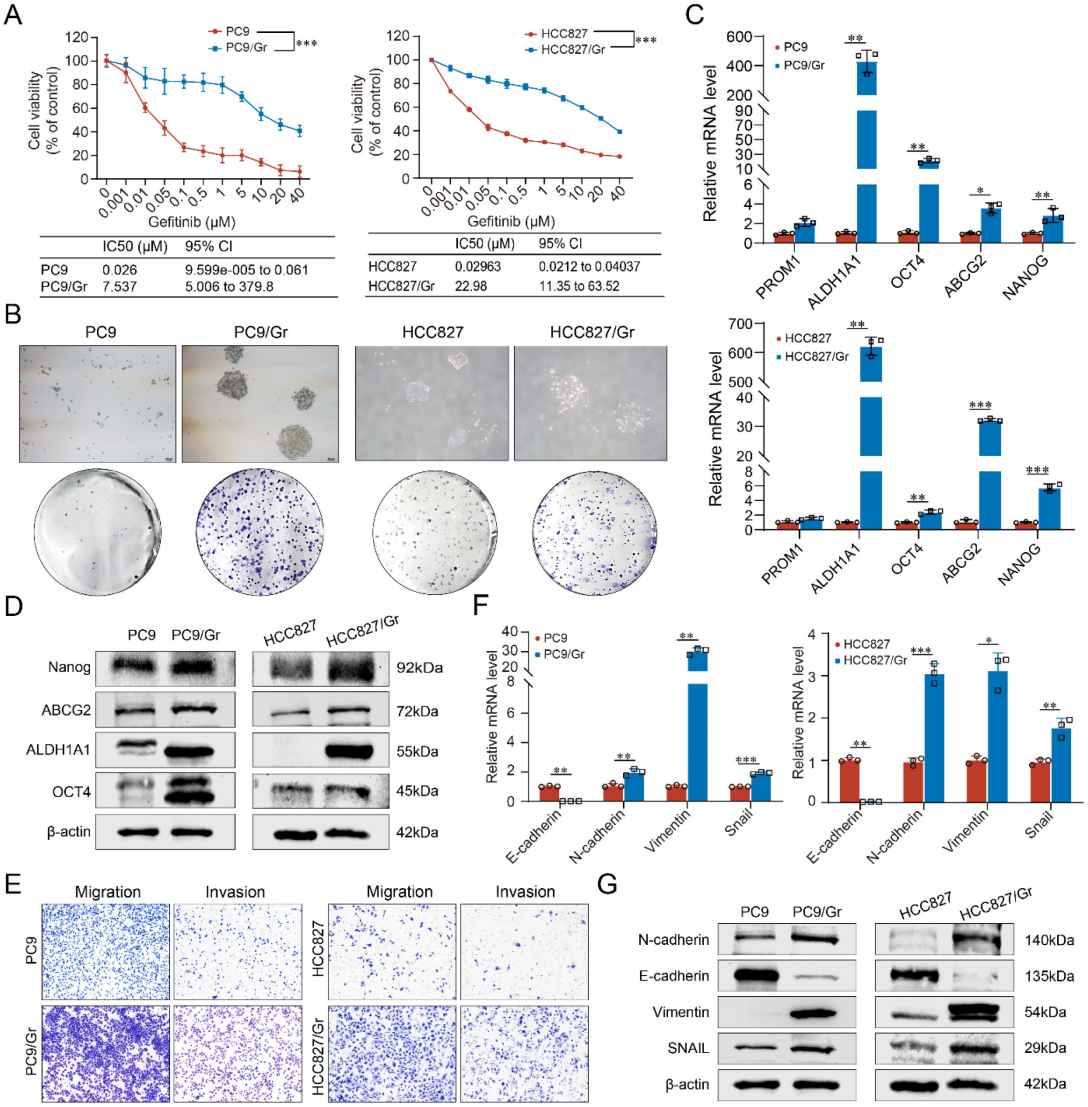
** The biological characterization of the gefitinib resistant lung cancer cells (PC9/Gr and HCC827/Gr). A.** Sensitivity to gefitinib in PC9 and PC9/Gr, HCC827 and HCC827/Gr cells.** B.** Images of tumor sphere formation in PC9 and PC9/Gr, HCC827 and HCC827/Gr cells (500 cells per well).** C.** CSC-related marker expression (PROM1, ALDH1A1, OCT4, ABCG2, and NANOG) in PC9 and PC9/Gr, HCC827 and HCC827/Gr cells detected by qRT-PCR.** D.** Western blot analysis of CSC-related markers in these cells.** E.** Transwell assay for migration and invasion abilities in PC9 and PC9/Gr, HCC827 and HCC827/Gr cells.** F.** EMT-related marker expression (E-cadherin, N-cadherin, Vimentin, and SNAIL) in PC9 and PC9/Gr, HCC827 and HCC827/Gr cells using qRT-PCR.** G.** Western blot analysis of EMT-related markers in these cells. *: *P* < 0.05; **:* P* < 0.01; ***: *P* < 0.001.

The parental lung cancer cells PC9 and HCC827 were exposed to gefitinib treatment for approximately 6 months to develop EGFR-TKIs-resistant lung cancer cells PC9/Gr and HCC827/Gr. The morphological characteristics of these gefitinib-resistant cells were examined using inverted microscopy. The PC9/Gr cells exhibited a flat and aggregated morphology, while the parental PC9 cells displayed a round or shuttle-shaped morphology, partially semi-adherent; however, both HCC827/Gr and HCC827 cells exhibited irregularly polygonal shapes, with the characteristic of aggregated growth more pronounced in HCC827/Gr cells, as shown in Supplementary Figure S1A. CCK8 assay showed that the IC50 of PC9/Gr and HCC827/Gr cells treated with gefitinib significantly increased compared to the corresponding parental cells, showing the drug resistance indices of 289.88 and 775.57 for PC9/Gr and HCC827/Gr cells, respectively (Figure 1A). In order to elucidate the potential mechanisms underlying gefitinib resistance in our established lung cancer cells, Sanger sequencing was performed and the results showed that *EGFR 19del* was present in these resistant lung cancer cells while there were no *EGFR T790M* mutation and other mutation such as *BRAF* or *PIK3CA* (Supplementary Figure S1B). Moreover, through qRT-PCR analysis, the *HER2* or *c-MET* amplifications were also not observed in these cells (Supplementary Figure S1C).

### Stemness phenotypic characteristics of gefitinib-resistant lung cancer cells

The possible mechanism of gefitinib resistance was investigated further to explore the stemness phenotypic characteristics in these cells. Enhanced self-renewal was observed *in vitro* in both PC9/Gr and HCC827/Gr cells (Figure 1B). Moreover, stemness genes, including *ABCG2*, *PROM1, ALDH1A1, OCT4*, and *NANOG*, were significantly elevated in the PC9/Gr and HCC827/Gr cells (Figure 1C and 1D and Supplementary Figure S2). Furthermore, these resistant cells demonstrated reduced sensitivity to the cytotoxic effects of chemotherapeutics, including cisplatin, pemetrexed, and gemcitabine (Supplementary Figure S3).

EMT (Epithelial-Mesenchymal Transition) represents another significant trait of stemness phenotype. The current study revealed that the PC9/Gr and HCC827/Gr cells exhibited enhanced migratory and invasive capabilities compared to the PC9 and HCC827 cells (Figure 1E). Consistently, the expression levels of EMT- associated genes, including N-cadherin, Vimentin, and Snail, were upregulated while the E-cadherin expression was downregulated in the PC9/Gr and HCC827/Gr cells (Figure 1F and 1G and Supplementary Figure S4).

### c-kit was involved in stemness and drug resistance in gefitinib-resistant lung cancer cells

**Figure 2:**
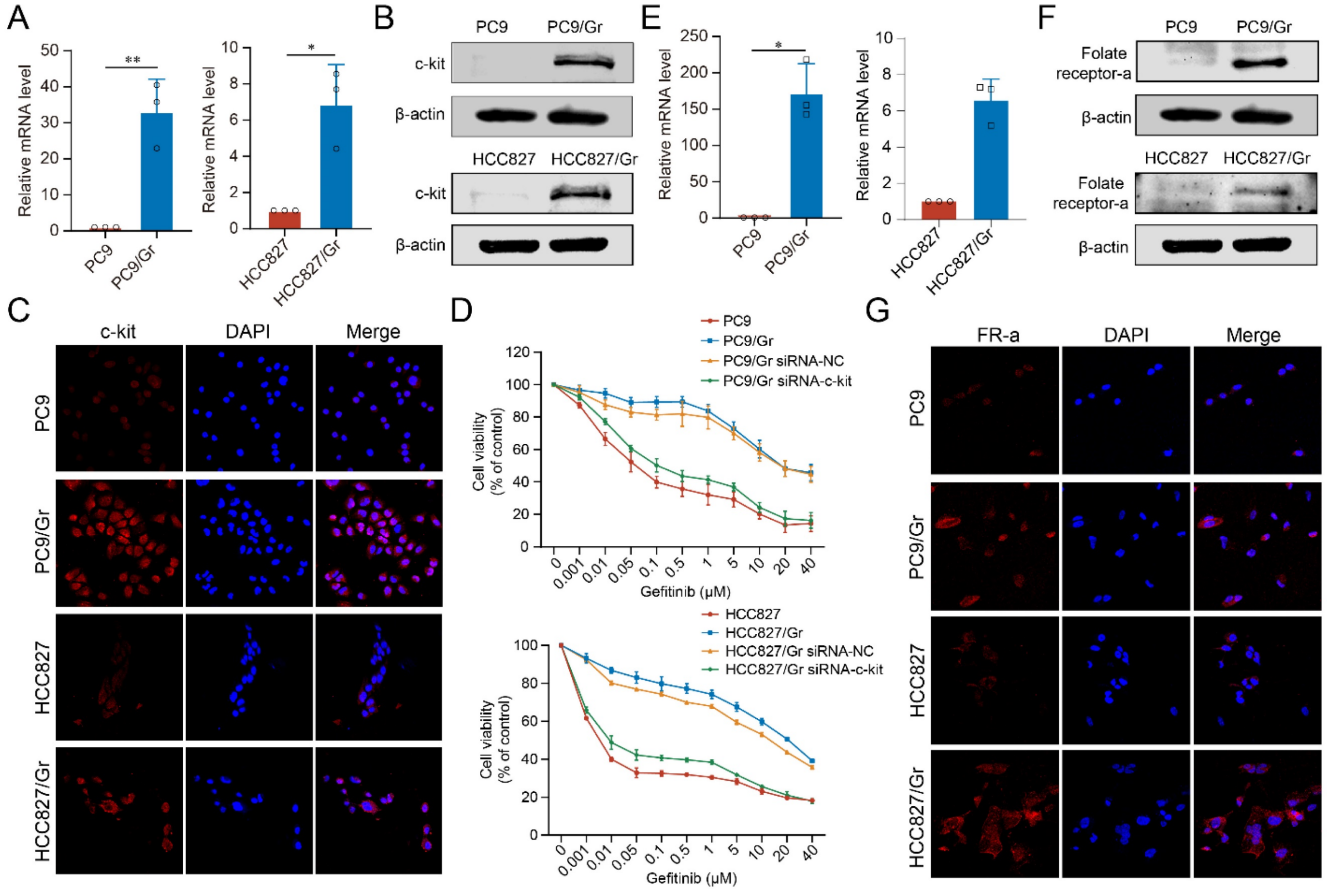
** Expression of c-kit and RNA interference in the gefitinib resistant lung cancer cells. A.** Increased mRNA expression of c-kit in PC9/Gr and HCC827/Gr cells compared to parental cells. **B.** Increased protein expression of c-kit in PC9/Gr and HCC827/Gr cells compared to parental cells. **C.** Immunofluorescence staining for c-kit in PC9 and PC9/Gr, HCC827 and HCC827/Gr cells. **D.** Increased sensitivity to gefitinib in PC9/Gr siRNA-c-kit and HCC827/Gr siRNA-c-kit cells compared to control cells. **E.** Increased mRNA expression of folate receptor-α in PC9/Gr and HCC827/Gr cells compared to parental cells. **F.** Increased protein expression of folate receptor-α in PC9/Gr and HCC827/Gr cells compared to parental cells. **G.** Immunofluorescence staining for folate receptor-α in PC9 and PC9/Gr, HCC827 and HCC827/Gr cells. *: *P* < 0.05; **:* P* < 0.01; ***: *P* < 0.001.

C-kit was found to be linked with proliferation, metastasis, and survival of cancer cells. The current study showed significantly increased expression levels of c-kit in both the PC9/Gr and HCC827/Gr cells compared to their respective parental cells (Figure 2A-2C). Our previous studies have demonstrated that c-kit was involved with stemness phenotypic maintaining in gefitinib resistant lung cancer cells 11. In this study, the interference capability of the siRNA-c-kit was validated (Supplementary Figure S5) and upon transfection into the PC9/Gr and HCC827/Gr cells, we observed a significant decrease in resistance to gefitinib (Figure 2D), which suggesting that c-kit might be served as a promising target for overcoming gefitinib resistance in lung cancer.

### Construction of FA-mExo-siRNA-c-kit

**Figure 3:**
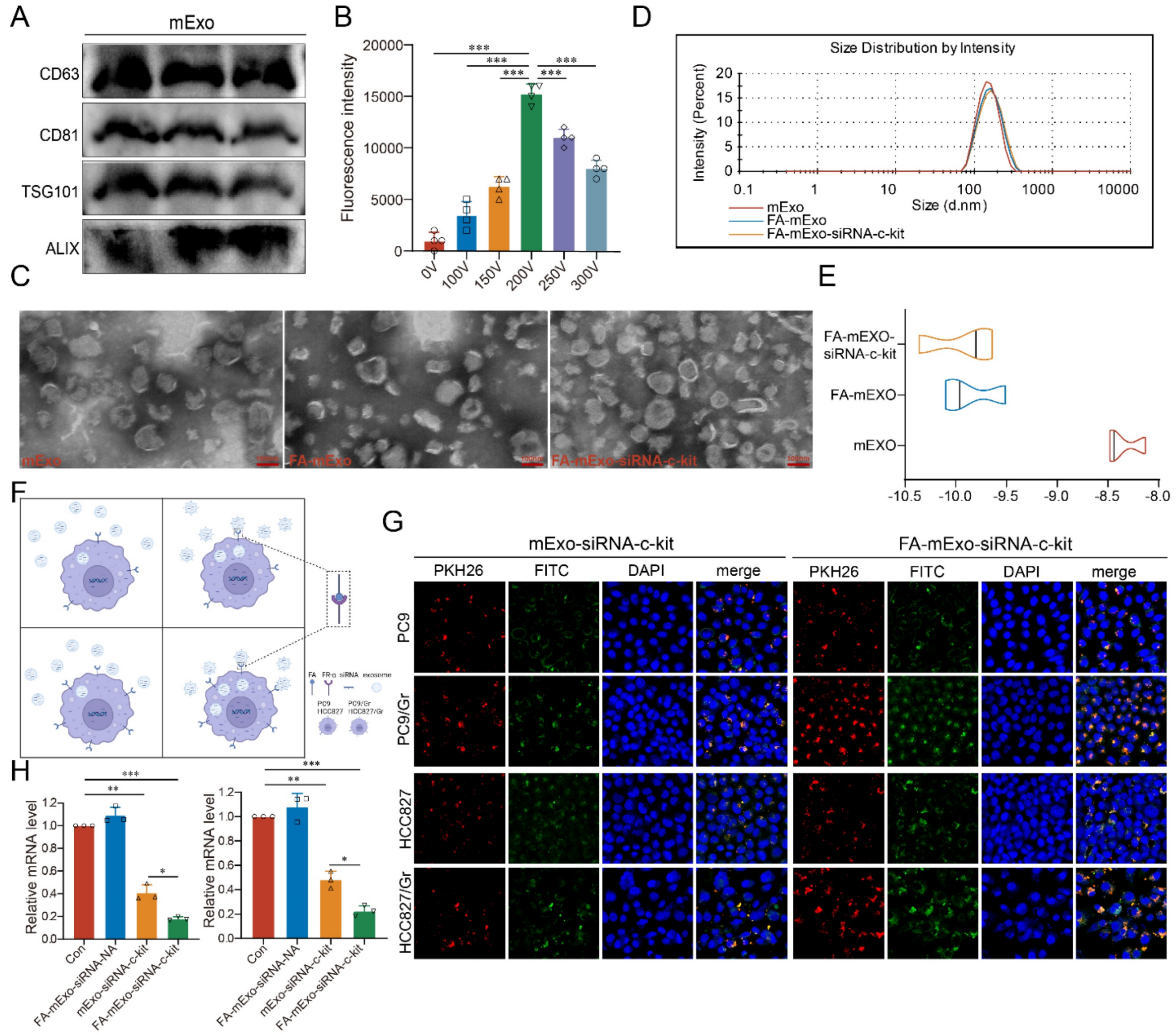
** Isolation, identification and modification of milk exosomes. A.** Evaluation of exosomal protein content in milk exosomes from three different batches. **B.** Detection of siRNA-c-kit fluorescence intensity in milk exosomes at varying electroporation voltages. **C.** TEM image of folic acid-modified milk exosomes loaded with siRNA. **D.** Nanoparticle tracking analysis of folic acid-modified milk exosomes loaded with siRNA. **E.** Zeta potential of folic acid-modified milk exosomes loaded with siRNA.** F.** Schematic plot of milk exosomes uptake by tumor cells. **G.** siRNA (green fluorescence) was loaded into milk exosomes (red fluorescence) by electroporation and applied on the PC9 and PC9/Gr, HCC827 and HCC827/Gr cells. After 24 h incubation cells were visualized by confocal microscope (scale 20 μm). **H.** siRNA against c-kit was loaded into milk exosomes and PC9/Gr and HCC827/Gr cells were treated for 48 h. Cells were collected for qPCR detection. *: *P* < 0.05; **:* P* < 0.01; ***: *P* < 0.001.

In order to develop efficient delivery vehicles for c-kit siRNA, milk exosomes (mExo) were extracted and purified using ultracentrifugation, followed by characterization of these exosomes using Western blot analysis. The analysis revealed that these exosomes expressed specific exosomal markers, including CD63, CD81, TSG101, and Alix, as shown in Figure 3A. While the successful isolation and identification of mExo were achieved, enhancing their targeting ability to cancer cells was crucial. In the current study, FA modification was employed due to the higher expression of FR-α in both the PC9/Gr and HCC827/Gr cells (Figure 2E-2G, and Supplementary Figure S6). The FA-mExo and mExo with PKH26 were labeled separately and co-incubated with PC9/Gr or HCC827/Gr cells to confirm the improved targetability of FA-modified mExo. The confocal microscopy images demonstrated that FA modification increased the uptake efficiency of gefitinib-resistant lung cancer cells towards mExo (Supplementary Figure S7). Moreover, the impact of FA on these cells was evaluated using CCK8 assay and the results showed that FA treatment did not affect the proliferation of PC9/Gr or HCC827/Gr cells (Supplementary Figure S8).

Subsequently, electroporation was employed to load siRNA-c-kit into mExo and generate FA-mExo-siRNA-c-kit. The optimization of siRNA loading into mExo involved adjusting voltage conditions to 10 ms pulse width and one pulse. FAM-labeled siRNA-c-kit (green) facilitated the determination of optimal voltage conditions. Using fluorescence microplate reader tests and confocal microscopy at various voltage conditions, it was confirmed that the maximum fluorescence intensity of FAM-siRNA-c-kit was achieved at 200 V, displaying perfect fluorescence overlap with mExo (red) at this voltage (Figure 3B and Supplementary Figure S9).

After constructing the FA-mExo-siRNA-c-kit, their characteristics were examined. TEM analysis revealed there was no discernible differences in morphology between FA-mExo-siRNA-c-kit and mExo (Figure 3C). However, a minor increase in the diameter of FA-mExo-siRNA-c-kit was observed in NTA (Nanoparticle Tracking Analysis) data (Figure 3D). Interestingly, the zeta potential of FA-mExo-siRNA-c-kit exhibited a significant decrease compared to mExo (Figure 3E) and we thought that this alteration resulted in a greater absolute value of surface charge, thereby enhancing exosome dispersion.

### Uptake of FA-mExo-siRNA-c-kit by gefitinib-resistant lung cancer cells

Subsequently, the ability of FA-mExo-siRNA-c-kit to target PC9/Gr and HCC827/Gr cells was assessed. Through fluorescence-labeling of FA-mExo-siRNA-c-kit and mExo-siRNA-c-kit, followed by co-incubation with lung cancer cells for 48 h, the highest fluorescent intensity was observed in gefitinib-resistant lung cancer cells treated with FA-mExo-siRNA-c-kit (Figure 3F and 3G). Furthermore, to validate the interference efficiency of FA-mExo-siRNA-c-kit, we measured the expression of the target gene c-kit in PC9/Gr or HCC827/Gr cells 48 hours after treatment with FA-mExo-siRNA-c-kit using qPCR. The results showed that FA-mExo-siRNA-c-kit treatment significantly reduced c-kit expression, with an interference efficiency of approximately 80% compared to the control group (Figure 3H).

### Biological activities of FA-mExo-siRNA-c-kit

**Figure 4:**
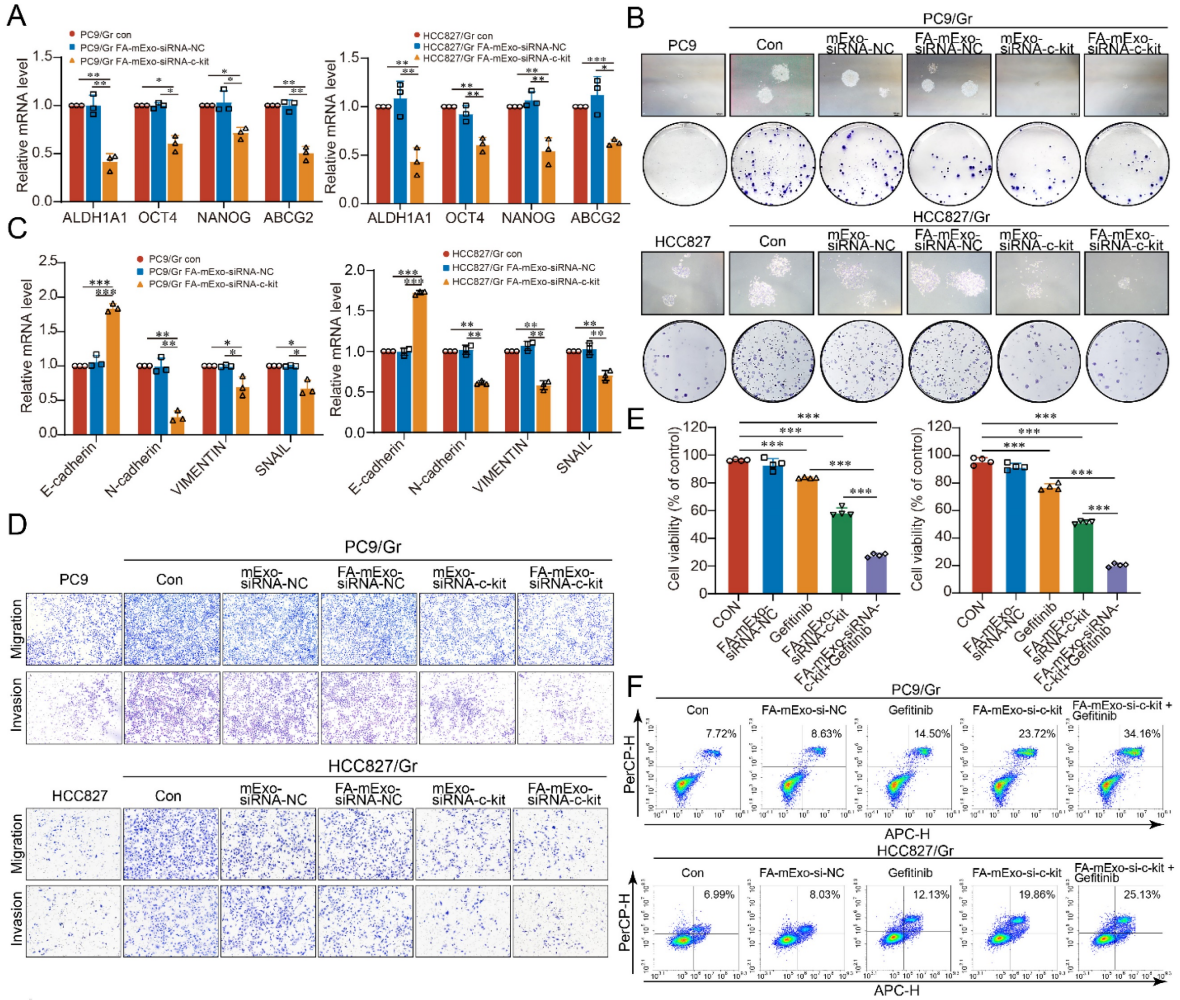
** Milk exosomes modified with folic acid and loaded with c-kit siRNA regulated the stemness phenotype of gefitinib resistant lung cancer cells and overcame gefitinib resistance. A.** The expression of stemness genes AHDL1A1, OCT4, NANOG and ABCG2 in PC9/Gr and HCC827/Gr cells were detected by qPCR after FA-mExo-siRNA-c-kit interference. **B.** The clonogenesis ability of PC9/Gr and HCC827/Gr cells treated with FA-mExo-siRNA c-kit was detected by clonal formation assay. **C.** The expression of EMT-related genes E-cadherin, N-cadherin, Vimentin and Snail in PC9/Gr and HCC827/Gr cells were detected by qPCR after FA-mExo-siRNA-c-kit treated. **D.** The migration and invasion ability of PC9/Gr and HCC827/Gr cells treated with FA-mExo-siRNA-c-kit were detected by transwell assay. **E.** The sensitivity of PC9/Gr and HCC827/Gr cells to gefitinib was increased after treatment with FA-mExo-siRNA-c-kit. **F.** Apoptosis assay showed that the number of apoptosis induced by gefitinib increased after FA-mExo-siRNA-c-kit treatment of PC9/Gr and HCC827/Gr cells. *: *P* < 0.05; **:* P* < 0.01; ***: *P* < 0.001.

The effects of these exosomes on the stemness phenotype of gefitinib-resistant lung cancer cells were assessed to further elucidate the biological activities of FA-mExo-siRNA-c-kit. Following treatment with FA-mExo-siRNA-c-kit, a significant decrease in the expression levels of *ABCG2*, *ALDH1A1*, *OCT4*, and *NANOG* was observed in both the PC9/Gr and HCC827/Gr cells (Figure 4A). Moreover, FA-mExo-siRNA-c-kit treatment inhibited the colony-forming ability of these cells (Figure 4B).

In order to assess the impact of FA-mExo-siRNA-c-kit on the EMT phenotype of these cells, the changes in EMT-related genes were first examined. The expression levels of N-cadherin, Vimentin, and Snail were downregulated, while that of E-cadherin was upregulated in FA-mExo-siRNA-c-kit-treated PC9/Gr and HCC827/Gr cells (Figure 4C). Moreover, the transwell assay revealed that FA-mExo-siRNA-c-kit treatment reduced the metastatic potential, including migration and invasion of PC9/Gr and HCC827/Gr cells (Figure 4D).

The effects of FA-mExo-siRNA-c-kit on gefitinib resistance were investigated further. The CCK8 assay demonstrated that the combination of gefitinib with FA-mExo-siRNA-c-kit significantly inhibited the proliferation of the PC9/Gr and HCC827/Gr cells compared to the gefitinib treatment alone (Figure 4E). Moreover, apoptosis assay revealed that FA-mExo-siRNA-c-kit treatment increased the sensitivity of PC9/Gr and HCC827/Gr cells to gefitinib (Figure 4F).

**Figure 5:**
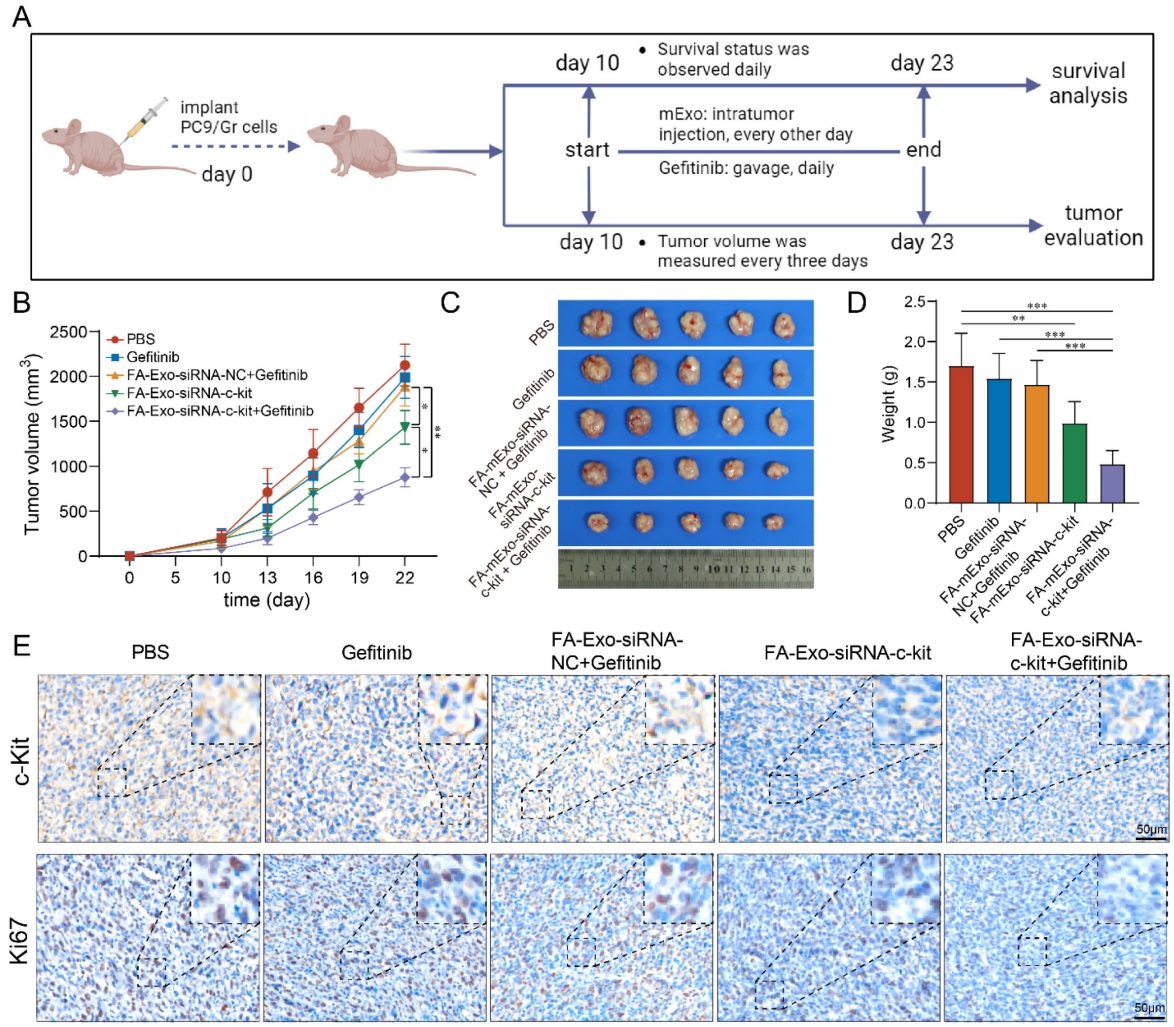
** The in vivo antitumor activities of FA-mExo-siRNA-c-kit. A.** Schematic diagram representing the subcutaneous xenografts mouse experiment to examine the relative tumor burden. **B.** Tumor volume of the mice with subcutaneous xenografts in various groups. **C.** Photographs of tumors harvested from mice with subcutaneous xenografts at day 23 from various groups. **D.** Tumor weight of the mice with subcutaneous xenografts at day 23 from various groups. **E.** Tumors were analyzed by IHC for c-Kit and Ki67 expression. Scale bar: 50 μm. *: *P* < 0.05; **:* P* < 0.01; ***: *P* < 0.001.

Subsequently, the biological effects of FA-mExo-siRNA-c-kit were examined* in vivo* (Figure 5A). Following the establishment of mice bearing subcutaneous PC9/Gr cell xenografts, the mice were divided into five groups: PBS group, gefitinib group, FA-mExo-siRNA-NC combined with gefitinib group, FA-mExo-siRNA-c-kit group, and FA-mExo-siRNA-c-kit combined with gefitinib group. The growth of subcutaneous xenografts was significantly suppressed in the FA-mExo-siRNA-c-kit combined with the gefitinib group compared to the gefitinib alone group (Figure 5B-5D). Additionally, immunohistochemical analysis revealed that FA-mExo-siRNA-c-kit successfully inhibited the expression of c-kit in gefitinib-resistant lung cancer xenografts, and the expression of Ki67 in the tumor tissues of the combined treatment group of mice was significantly reduced (Figure 5E).

### Biodistribution and toxicity of FA-mExo-siRNA-c-kit in vivo

**Figure 6:**
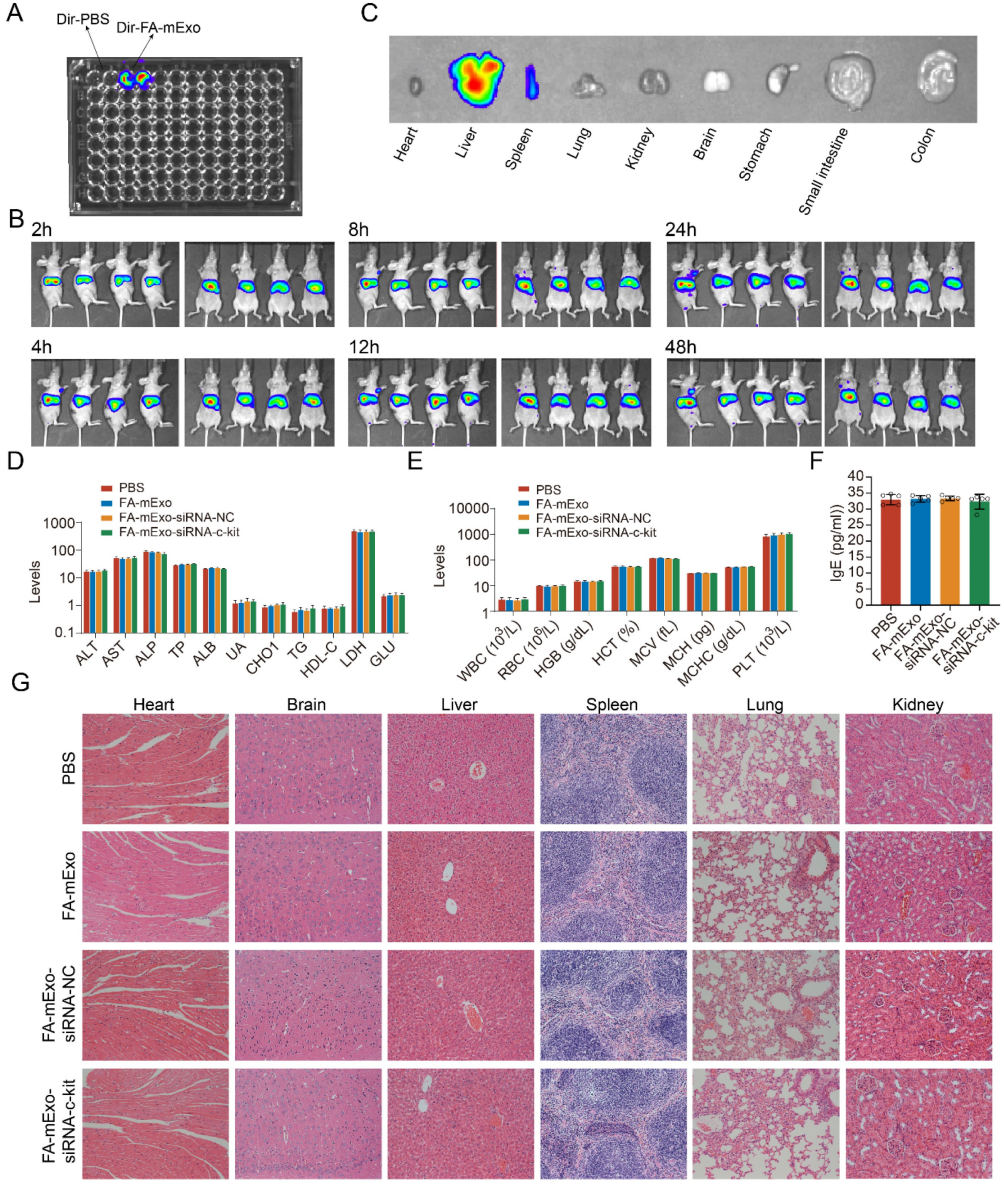
** Biological distribution and safety analysis of FA-mExo-siRNA-c-kit after vein injection. A.** DiR-labelled mExo and PBS control were subjected to fluorescent imaging using IVIS in a 96-well plate. DiR-labelled mExo showed a strong fluorescent signal as opposed to no signal in the control treated with PBS. **B.** In vivo fluorescence imaging of milk exosomes at different time points after intravenous injection. **C.** The column shows ex vivo fluorescence images of major organs and of a liver metastasis of lung cancer harvested from those mice at 24 h after intravenous injection. **D.** Blood cell analysis, liver and kidney function enzymes (**E**) levels were measured after FA-mExo-siRNA-c-kit treatment compared with control treatment. **F.** Serum IgE levels were measured after FA-mExo-siRNA-c-kit treatment compared with control treatment. **G.** H&E stained heart, brain, liver, spleen, lung, and kidney specimens harvested from the treated mice after two weeks of treatment. *: *P* < 0.05; **:* P* < 0.01; ***: *P* < 0.001.

Considering the challenging prognosis faced by lung cancer patients with liver metastasis, liver metastasis was induced in BALB/c nude mice using gefitinib-resistant lung cancer cells to evaluate the efficacy of anti-tumor interventions. Initially, the biodistribution of these exosomes was assessed. The FA-mExo-siRNA-c-kit was labeled with the Dir fluorophore (Dir-FA-mExo-siRNA-c-kit), and its biodistribution was monitored using IVIS (Figure 6A). After the administration of Dir-FA-mExo-siRNA-c-kit via the tail vein, substantial enrichment in the liver was observed, persisting for approximately 48 hours (Figure 6B). The mice were euthanized to confirm liver enrichment by observing the fluorescent intensity in vital organs. As shown in Figure 6C, Dir-FA-mExo-siRNA-c-kit predominantly accumulated in the liver, likely attributing to its' crucial roles in drug metabolism.

The *in vivo* toxic potential of FA-mExo-siRNA-c-kit was determined by administering it to wild-type C57BL/6 mice via tail vein injection for one week, during which their behavior and diet were monitored (data not shown). No significant changes were observed in these parameters. The hematological and serum biochemical parameters related to liver and kidney function were assessed to evaluate systemic toxicological effects. The results showed no substantial differences in these data following FA-mExo-siRNA-c-kit treatment (Figure 6D and 6E). Notably, given that mExo is allogenic and might elicit allergic reactions upon tail vein injection in wild-type C57BL/6 mice, the hypersensitivity index IgE was measured using ELISA. The findings indicated that FA-mExo-siRNA-c-kit treatment did not induce significant changes in IgE levels in these mice (Figure 6F). Moreover, the vital organs were examined by staining with H&E, revealing no pathological structural changes in the heart, liver, spleen, lung, kidney and brain, respectively (Figure 6G).

### Regulation of gefitinib resistance of FA-mExo-siRNA-c-kit in liver metastasis model

**Figure 7:**
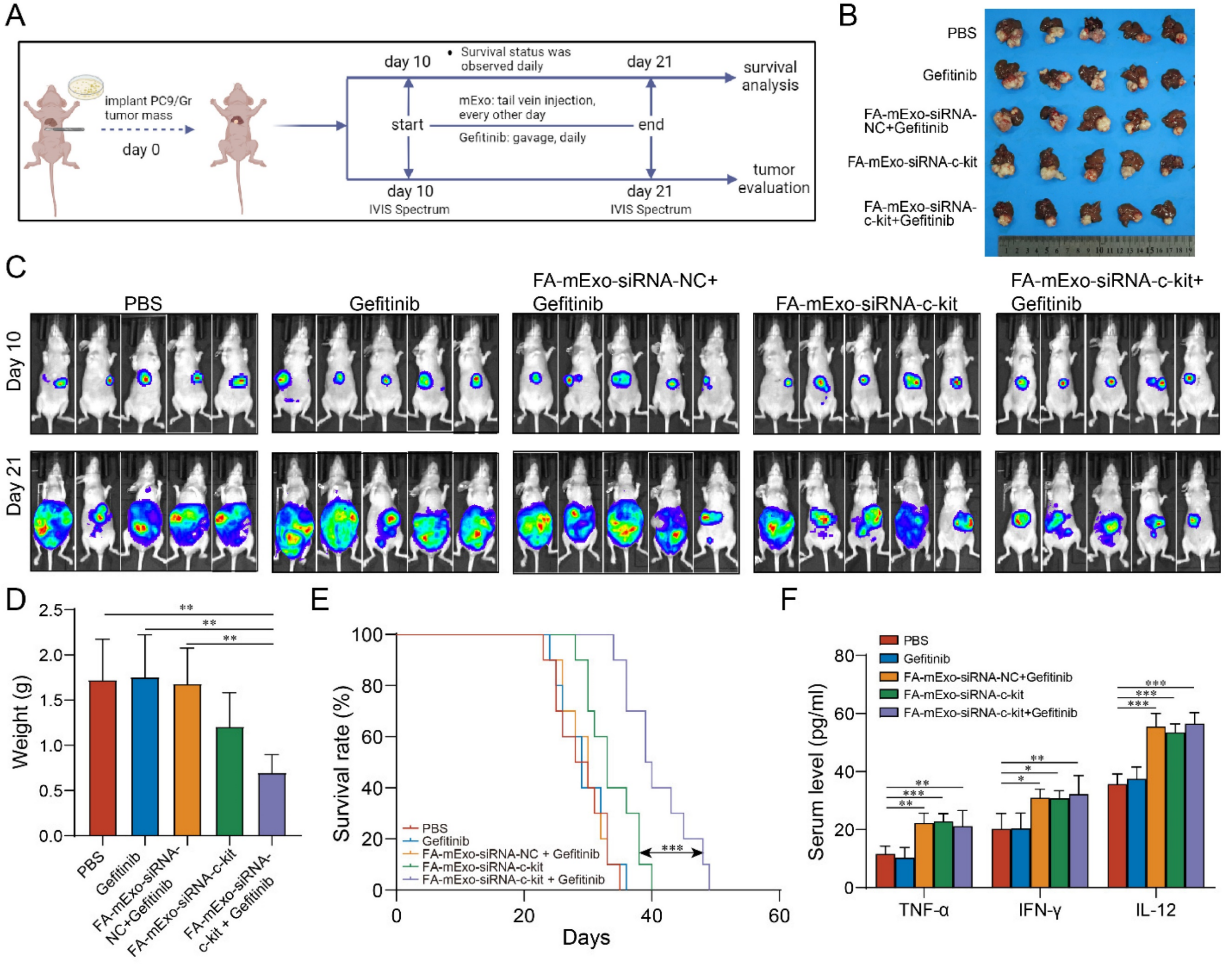
** The antitumor activities of FA-mExo-siRNA-c-kit in liver metastasis model. A.** Schematic diagram representing the liver metastases mouse experiment to examine the relative tumor burden. **B.** Photographs of liver metastases harvested from mice at day 24 from various groups. **C.** In vivo imaging analysis of liver metastasis model of drug-resistant lung cancer in different treatment groups. **D.** Tumor weight of the mice with liver metastases at day 24 from various groups. **E.** Kaplan-Meier curve was used to analyze the survival of the liver metastases mouse in each treatment group. **F.** ELISA of serum TNF-α, IFN-γ and IL-12 determination. *: *P* < 0.05; **:* P* < 0.01; ***: *P* < 0.001.

Based on the biological distribution and safety profile of FA-mExo-siRNA-c-kit administered via tail vein injection, its biological effects were further evaluated in the liver metastasis model (Figure 7A). The liver metastasis BALB/c nude mice were divided into five groups: PBS group, gefitinib group, FA-mExo-siRNA-NC combined with gefitinib group, FA-mExo-siRNA-c-kit group, and FA-mExo-siRNA-c-kit combined with gefitinib group. Following treatment, the results revealed a significant inhibition in the growth of liver metastases in the FA-mExo-siRNA-c-kit combined with the gefitinib group compared to the gefitinib group (Figure 7B-7D). Kaplan-Meier survival curve analysis further demonstrated a significantly prolonged survival time of mice in the FA-mExo-siRNA-c-kit combined with gefitinib group compared to the PBS, gefitinib, FA-mExo-siRNA-c-kit, and FA-mExo-siRNA-NC combined with gefitinib groups, respectively (Figure 7E).

Previous studies indicated that liver metastasis decreased the effects of immune checkpoint inhibitors via exacerbating immunosuppressive microenvironment in liver 32-36. In this study, we explored the possible impact of milk exosomes treated groups on the immune microenvironment in liver metastasis. Through detecting the cytokines levels using ELISA analysis, we found that, compared to the control group, the mExo-treated groups exhibited significantly elevated serum concentrations of cytokines TNF-α, IFN-γ, and IL-12, suggesting that mExo might enhance the innate immune response against tumor cells (Figure 7F).

### Gefitinib resistance was involved in the stemness phenotype transformation via the c-kit/mTOR pathway

**Figure 8:**
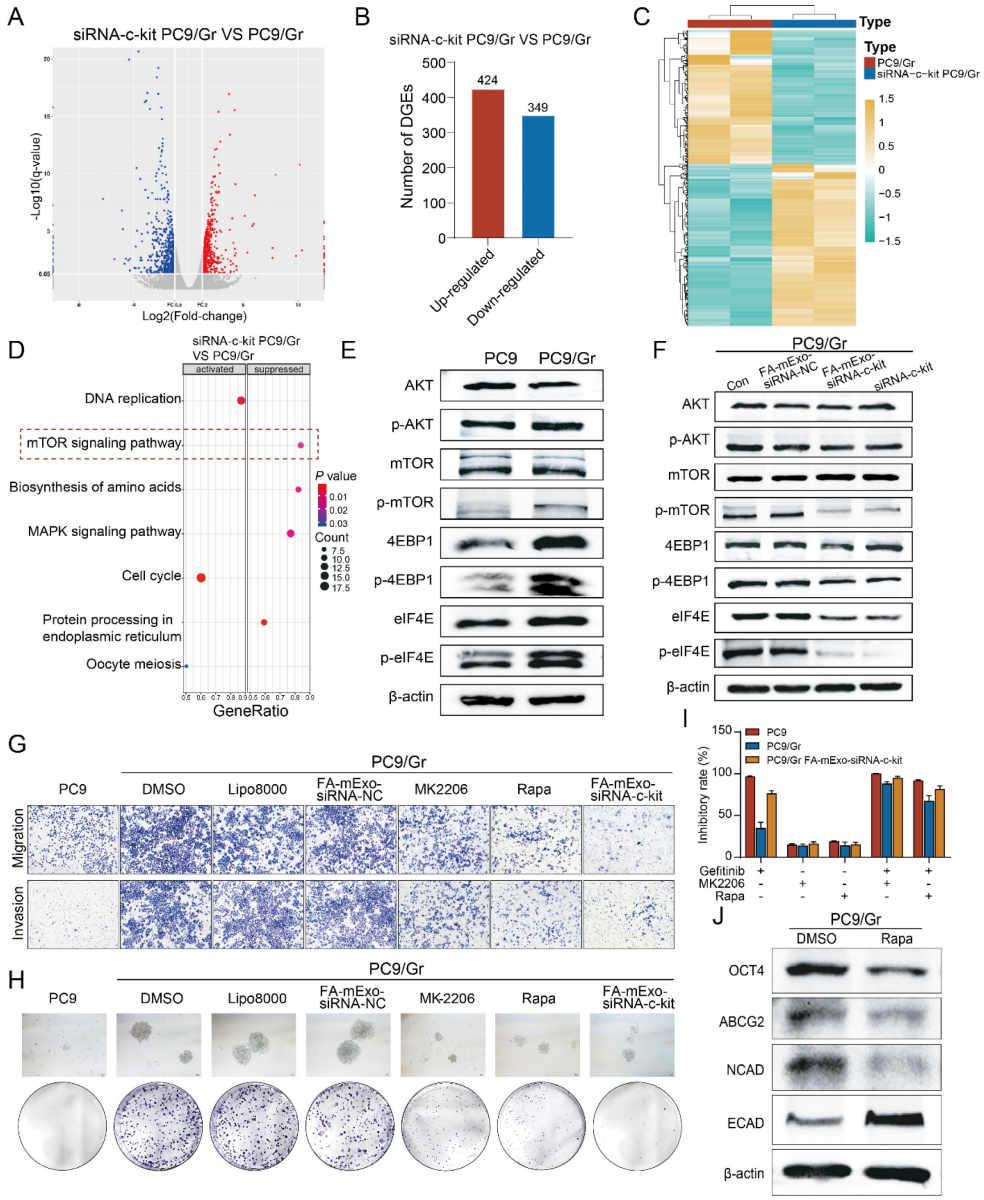
** FA-mExo-siRNA-c-kit targeted inhibition of mTOR pathway overcomes gefitinib resistance caused by stemness transformation. A.** Volcanic maps showing differential genes in mRNA sequencing of PC9/Gr and siRNA-c-kit PC9/Gr cells. **B.** Statistical analysis of differential genes in mRNA sequencing of PC9/Gr and siRNA-c-kit PC9/Gr cells. **C.** Heatmap showing differential genes in mRNA sequencing of PC9/Gr and siRNA-c-kit PC9/Gr cells. **D.** GSEA analysis of differential genes in mRNA sequencing of PC9/Gr and siRNA-c-kit PC9/Gr cells. **E.** Protein expression of mTOR signaling pathway-related molecules AKT, p-AKT, mTOR, p-mTOR, 4EBP1, p-4EBP1, eIF4E, p-eIF4E in PC9 and PC9/Gr cells. **F.** Suppression of mTOR signaling pathway after interference of c-kit expression in PC9/Gr cells. **G.** The migration and invasive abilities of PC9/Gr cells were assessed with transwell assays after treatment with MK2206 (AKT inhibitor) or rapamycin (mTOR inhibitor). **H.** The sphere formation efficiency of PC9/Gr cells was evaluated after treatment with MK2206 (AKT inhibitor) or rapamycin (mTOR inhibitor). **I.** Effects of AKT/mTOR inhibitors on gefitinib resistance after blocking the mTOR signaling pathway in PC9/Gr cells.** J.** The expression of stem cell-related genes OCT4, ABCG2 and mesenchymal marker N-cadherin was diminished by MK2206 and rapamycin treatment, and epithelial makers E-cadherin expression was increased in PC9/Gr cells. *: *P* < 0.05; **:* P* < 0.01; ***: *P* < 0.001.

The current study demonstrated the efficacy of FA-mExo siRNA-c-kit in overcoming gefitinib resistance both *in vitro* and *in vivo*, primarily by attenuating stemness features, including the reversal of EMT and reduction of *ABCG2* expression levels in gefitinib-resistant lung cancer cells. In order to elucidate the underlying mechanism, RNA-seq was performed in gefitinib-resistant lung cancer cells after transfection with c-kit siRNA, establishing PC9/Gr vector and PC9/Gr c-kit siRNA groups. The findings revealed 424 upregulated and 349 downregulated genes in the PC9/Gr c-kit siRNA group compared to the PC9/Gr vector group (Figure 8A-8C). Subsequent analysis using GSEA (Gene Set Enrichment Analysis) indicated a significant suppression in the mTOR signaling pathway upon downregulation of c-kit expression (Figure 8D), a pathway well-known for its involvement in the transformation of stemness phenotype.

Given the significant enrichment of downstream molecules, such as 4EBP1 and eIF4E, in the mTOR pathway, their expression levels were examined. Western blot analysis confirmed significantly higher phosphorylation levels of AKT, mTOR, 4EBP1, and eIF4E in both the PC9/Gr and HCC827/Gr cells compared to the PC9 and HCC827 cells (Figure 8E and Supplementary Figure S10A). Furthermore, reducing c-kit expression through either mExo or lipo8000-mediated delivery of c-kit siRNA decreased the phosphorylation levels of AKT, mTOR, 4EBP1, and eIF4E in the PC9/Gr and HCC827/Gr cells (Figure 8F and Supplementary Figure S10B).

Furthermore, pharmacological inhibitors, MK2206 (an AKT inhibitor) and rapamycin (mTOR inhibitor) were used, both of which are pivotal regulators in the mTOR signaling pathway. These inhibitors effectively suppressed colony formation, migration, and invasion of gefitinib-resistant lung cancer cells (Figure 8G and 8H and Supplementary Figure S10C and S10D). Moreover, MK2206 and rapamycin enhanced the inhibitory effects of gefitinib on cell proliferation in the PC9/Gr and HCC827/Gr cells, respectively (Figure 8I and Supplementary Figure S10E). Notably, they downregulated the expression levels of stemness and mesenchymal-related genes, including *OCT4*, *ABCG2*, and *N-cadherin*, while upregulating those of the epithelial-related gene *E-cadherin* (Figure 8J and Supplementary Figure S10F).

## Discussion

EGFR-TKIs have demonstrated efficacy in treating lung cancer patients with EGFR mutations. However, the emergence of TKI resistance, especially progressed with liver metastasis, faces a significant challenge, necessitating the investigation of novel resistance mechanisms and the development of effective therapeutic strategies. Secondary mutations in the EGFR driver gene are a primary cause of resistance to EGFR-TKIs 2, 3; in addition, other mutations including *BRAF*, *PIK3CA, HER2* or *c-MET* amplifications, which were independent of EGFR pathway, account for 5-20% in EGFR-TKIs resistant lung cancer 37. In the present study, we detected and found that there was no T790M mutation or other mutations including *BRAF*, *PIK3CA, HER2* or *c-MET* amplifications in these resistant lung cancer cells. Interestingly, *EGFR 19del* mutation was present in these resistant cells, which indicated that there might be some other reason initiating gefitinib resistance in these lung cancer cells. Histological type transformation such as small cell transformation in EGFR-TKIs resistant lung cancer is another mechanism, which is involved with persistent presence of *EGFR 19del* mutation and new mutations such as TP53 and Rb1. In this process, the growth of resistant lung cancer cells was initiated by small cell lung cancer and sensitive to the cytotoxicity of cisplatin and etoposide chemotherapy 38. Combined with the evidences in the present study that our established resistant lung cancer cells were endowed with stemness phenotypic characteristics including enhanced colony-forming ability and epithelial-to-mesenchymal transition (EMT) phenotype as well as upregulated expression levels of stemness-related genes, including *ABCG2*, *CD133*, and *Oct4*, we thought that, in spite of the presence of *EGFR 19del* mutation in these resistant cells, at this time, stemness might initiate the growth of these cells and exploring the possible target which regulated the stemness in these cells might offer a promising approach to overcoming gefitinib resistance in these cells.

C-kit, a crucial member of the tyrosine kinase family, is predominantly expressed in various tumor types, including gastrointestinal tumors, acute myeloid leukemia, and melanoma 39-41. Previous reports demonstrated that c-kit was closely involved with stemness phenotype maintaining in cancer stem cells 42. Koran S* et al.* 43 reported that CD117/c-kit expression in circulating tumor cells of advanced prostate cancer patients is associated with increased aggressiveness, decreased survival, and resistance to tyrosine kinase inhibitors by promoting cancer stem cell properties. Besides, Fang C *et al.* 44 found that the c-Kit/PHB axis enhances ovarian cancer stemness, tumorigenicity, and chemotherapy resistance by promoting PHB phosphorylation and stabilizing Notch3 and β-catenin signaling pathways, suggesting it as a potential therapeutic target. Thus, targeting c-kit expression in these cells might offer a promising approach to overcome drug resistance. Indeed, in our study, we verified that targeting c-kit attenuated stemness of gefitinib resistant lung cancer cells and sensitized these cells to gefitinib treatment due to the persistent presence of *EGFR 19del* mutation.

While RNA interference technology, including siRNA, has emerged as a pivotal approach in cancer-targeted therapy, its *in-vivo* delivery remains challenging due to its susceptibility to degradation and off-target effects. Exosomes, natural nanoscale extracellular vesicles, offer a promising solution to these obstacles. Previous studies indicated that exosomes derived from cancer cells carrying siRNA-S100A4 could effectively protect siRNA from degradation, leading to significant suppression of cancer cells 45. Milk exosomes possess stability, biocompatibility, and low immunogenicity 30, and have unique advantages. They are abundant, cost-effective, and easily accessible, with higher concentrations compared to exosomes from mesenchymal stem cells, HEK-293T cells, and other cultured cells 20. Unlike exosomes from other cells, which may promote cancer cell proliferation and invasion, milk exosomes enhance immune responses, making them an ideal siRNA delivery vehicle with significant clinical potential in cancer therapy 46. In the present study, we constructed FA-mExo-siRNA-c-kit and firstly verified the targetability to gefitinib resistant lung cancer cells and the anti-tumor effects including decreasing stemness and inducing apoptosis *in vitro*.

Due to the livers' frequent involvement as a metastatic site in lung cancer, the emergence of liver metastasis after acquiring resistance to EGFR-TKIs is very common 4. Liver metastases are characterized by a distinct tumor microenvironment that often confers resistance to conventional therapies, including chemotherapy, targeted therapy, and immunotherapy, thereby contributing to poor patient prognosis 5. The current study observed a significant accumulation of FA-mExo in the liver and liver metastases following intravenous administration in mice, consistent with previous studies 47. Therefore, the anti-tumor efficacy of FA-mExo-c-kit siRNA in combination with gefitinib in drug-resistant lung cancer with liver metastases was explored. After establishing orthotopic liver metastasis xenograft mouse models in gefitinib resistant lung cancer, we demonstrated that the combination treatment significantly suppressed the growth of gefitinib-resistant lung cancer in liver metastases compared to gefitinib monotherapy. This result suggested that FA-mExo-siRNA-c-kit might be effective in treating gefitinib-resistant lung cancer with liver metastases.

Immunotherapy, particularly immune checkpoint inhibitors (ICIs), has brought significant hope to advanced cancer patients, but not all patients benefit from it. Clinical data indicate that patients with liver metastasis typically exhibit poorer responses to ICIs compared to those without liver involvement, likely due to the liver's immune-exempt status and its highly immunosuppressive microenvironment, which facilitates immune evasion 32-36. Studies suggest that milk exosomes play a beneficial role in modulating the tumor microenvironment by enhancing immune responses and regulating immune cells to inhibit tumor growth and metastasis 48. Therefore, harnessing the unique advantages of milk exosomes holds promise for advancing immune-based therapies against lung cancer and its liver metastases. In this study, we detected the immune microenvironment in liver metastases of gefitinib-resistant lung cancer. Our results preliminary revealed that milk exosomes might increase the level of TNF-α, IFN-γ, and IL-12 *in vivo*, which was closely associated with the innate immune response against cancer cells. Since they are nude mice, we cannot thoroughly investigate changes in the immune microenvironment, but these findings suggest that milk exosomes might collaborate with or enhance the immune system and provide a basis for future combination strategies with immunotherapy.

In order to elucidate the molecular mechanism by which c-kit modulated the stemness phenotype to overcome gefitinib resistance, RNA-seq analysis of gefitinib-resistant lung cancer cells was conducted following transfection with c-kit siRNA. The results revealed a significant suppression of the mTOR signaling pathway in these cells. The mTOR signaling pathway is well-established in cancer biology, orchestrating vital functions, including survival, apoptosis, angiogenesis, and autophagy through phosphorylation-mediated inactivation of the translational repressors, including eukaryotic initiation factor 4E (eIF4E) and 4E binding protein 1 (4EBP1). The current study found elevated phosphorylation levels of eIF4E and 4EBP1 in gefitinib-resistant lung cancer cells, which declined upon the downregulation of c-kit expression. This implied that c-kit could regulate the stemness phenotype transformation in gefitinib-resistant lung cancer cells through the AKT/mTOR/4EBP1/eIF4E axis. Recent studies have shown the association of the mTOR signaling pathway with stemness phenotype transformation and drug resistance across diverse cancers, such as breast cancer, colorectal cancer, and glioblastoma 49, 50. The current study demonstrated that the AKT inhibitor MK2206 or the mTOR inhibitor rapamycin could mitigate the stemness of gefitinib-resistant lung cancer cells and enhance their sensitivity to gefitinib by suppressing the mTOR signaling pathway. Therefore, it was proposed that the role of c-kit in regulating the stemness phenotype to overcome gefitinib resistance might be intricately linked to the mTOR/4EBP1/eIF4E signaling axis. This represented a novel mechanism underlying EGFR-TKIs resistance in lung cancer.

Despite the promising findings of the current study, several limitations need to be addressed to optimize the therapeutic strategy for overcoming EGFR-TKI resistance in lung cancer. Firstly, the efficiency of milk exosomes encapsulating drugs or biomacromolecules is relatively low, which may be related to the inherent characteristics of the carrier and the encapsulation techniques. Recent advancements in exosome engineering, such as removing endogenous content through extrusion, freeze-thaw cycles, or chemical permeabilization, have shown potential to enhance encapsulation efficiency 51, 52. Additionally, while folic acid modification of exosomes provides a degree of targeting specificity due to the overexpression of folate receptors in EGFR-TKI resistant lung cancer cells, more precise targeting strategies should be explored. Future research should focus on improving the efficiency of drug or biomacromolecule encapsulation in milk exosomes and exploring more precise targeting strategies. Furthermore, safety assessment is a key aspect of this study. While we have paid special attention to and preliminarily verified its biosafety, more in-depth safety evaluations and validations are necessary for clinical translation. Addressing these challenges will be critical for facilitating the clinical translation of this promising therapeutic strategy, ultimately improving patient outcomes in lung cancer treatment.

## Conclusions

**Figure 9:**
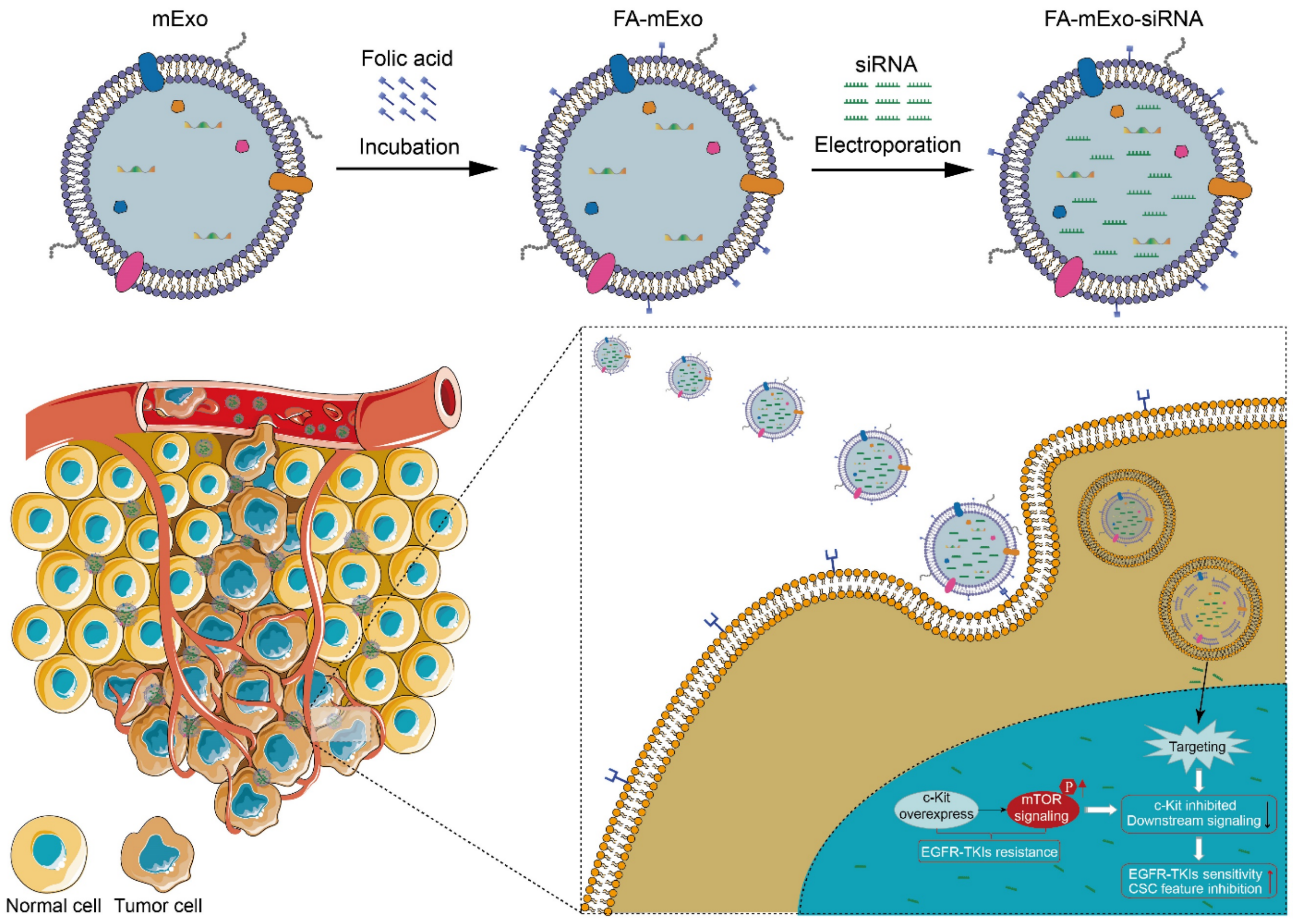
**Flowchart of the study.** Folic acid-modified milk exosomes carrying c-kit siRNA targets EGFR-TKIs resistant lung cancer cells and interferes with c-kit expression, thereby suppressing mTOR signaling-driven stemness phenotype transformation to overcome targeted therapy resistance.

The current study demonstrated the efficacy of FA-modified milk exosomes carrying c-kit siRNA in overcoming gefitinib resistance in lung cancer both *in vitro* and *in vivo* by attenuating stemness. This effect appeared to be associated with the downregulation of *ABCG2* expression levels and reversal of the EMT phenotype. The molecular investigations indicated that c-kit regulated the AKT/mTOR/4EBP1/eIF4E axis, thus driving the transformation of the stemness phenotype in gefitinib-resistant lung cancer cells (Figure 9). Briefly, the findings of the current study shed light on a novel molecular mechanism underlying EGFR-TKI-resistant lung cancer, presenting a promising therapeutic strategy. Further research is needed to facilitate the clinical translation of these findings for the benefit of lung cancer patients, especially in immune microenvironment and liver metastasis.

## Supplementary Material

Supplementary figures and table.
