# Supplementary material for: Folic Acid-Modified Milk Exosomes Delivering c-Kit siRNA Overcome EGFR-TKIs Resistance in Lung Cancer by Suppressing mTOR Signaling and Stemness
Source: Int J Biol Sci. 2025 Jan 1;21(1):382–99. doi: 10.7150/ijbs.99954 (PMC11667802; doi:10.7150/ijbs.99954)
Supplement: Supplementary file 1 — Supplementary figures and table. [file ijbsv21p0382s1.pdf]

Supporting Information

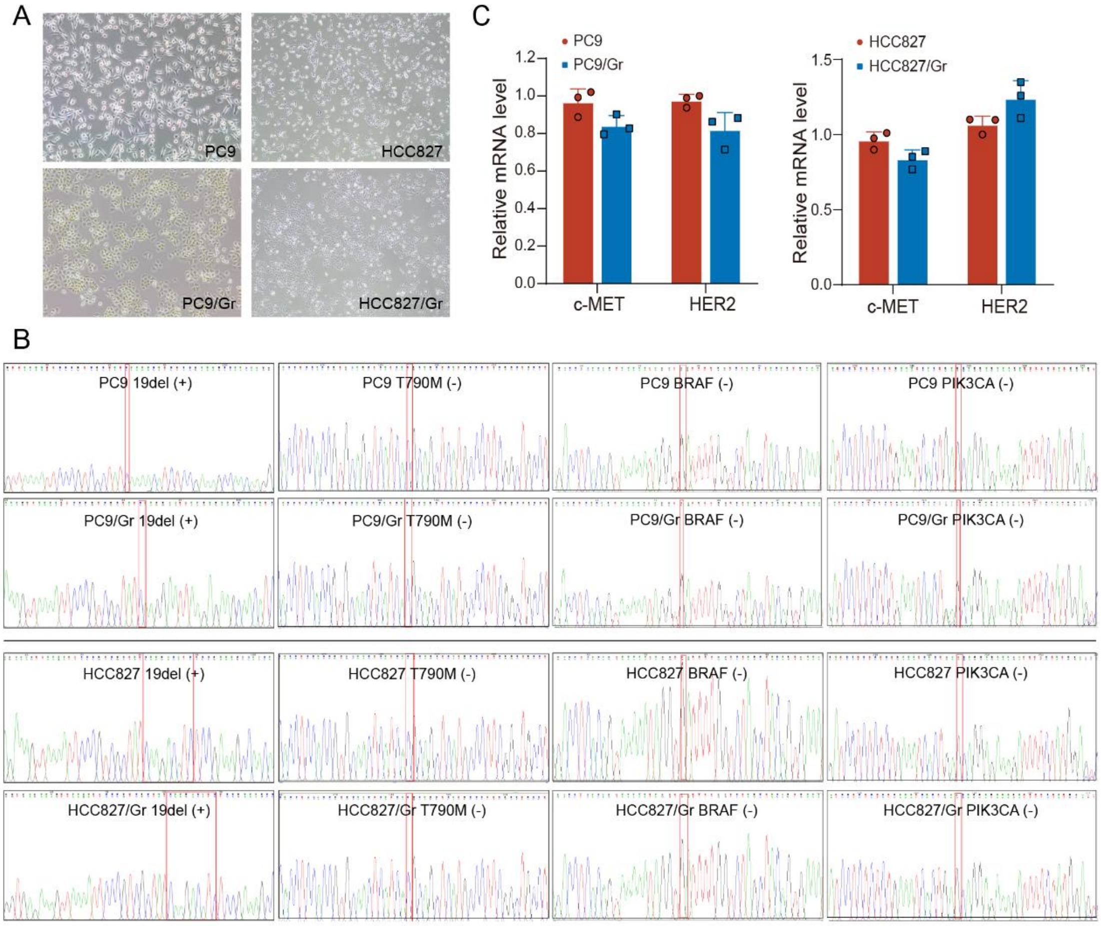

Supplementary Figure S1. A. The morphology of lung cancer cells (PC9 and HCC827) and gefitinib resistant lung cancer cells (PC9/Gr and HCC827/Gr). B. Sanger sequencing for EGFR T790M, BRAF and PIK3CA of PC9 and PC9/Gr, HCC827 and HCC827/Gr cells. C. The expression of C-MET and HER2 in PC9 and PC9/Gr, HCC827 and HCC827/Gr cells was detected by qRT-PCR.

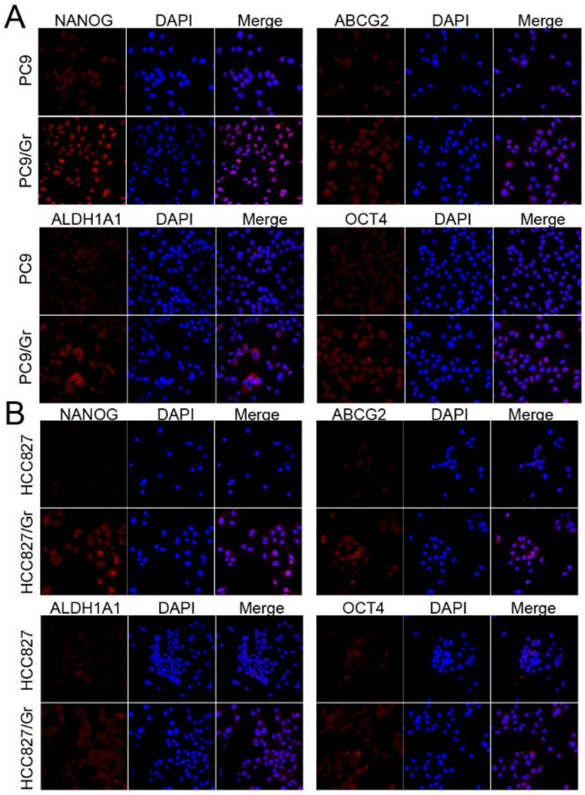

Supplementary Figure S2. A. Immunofluorescence staining of stemness related genes NANOG, ABCG2, ALDH1A1 and OCT4 in PC9 and PC9/Gr cells. B. Immunofluorescence staining of stemness related genes NANOG, ABCG2, ALDH1A1 and OCT4 in HCC827 and HCC827/Gr cells.

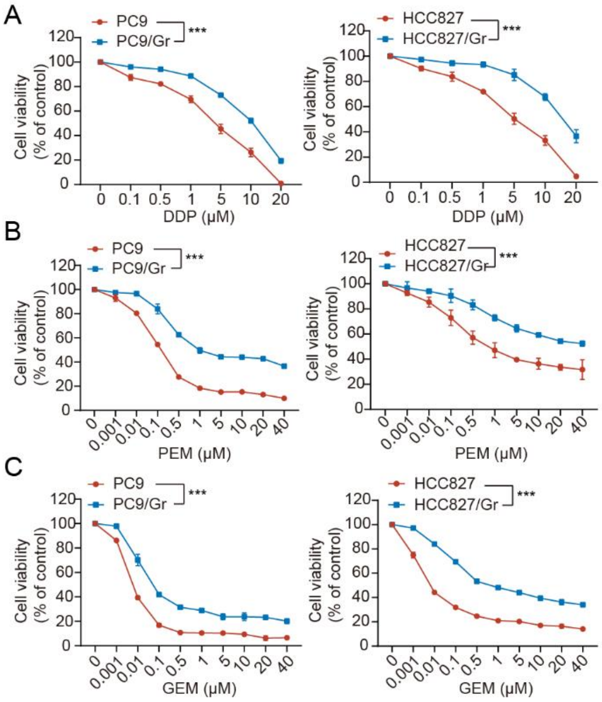

Supplementary Figure S3. The sensitivities of PC9 and PC9/Gr, HCC827 and HCC827/Gr cells to cisplatin (A), pemetrexed (B), and gemcitabine (C). *: $P < 0.05$; **: $P < 0.01$; ***: $P < 0.001$.

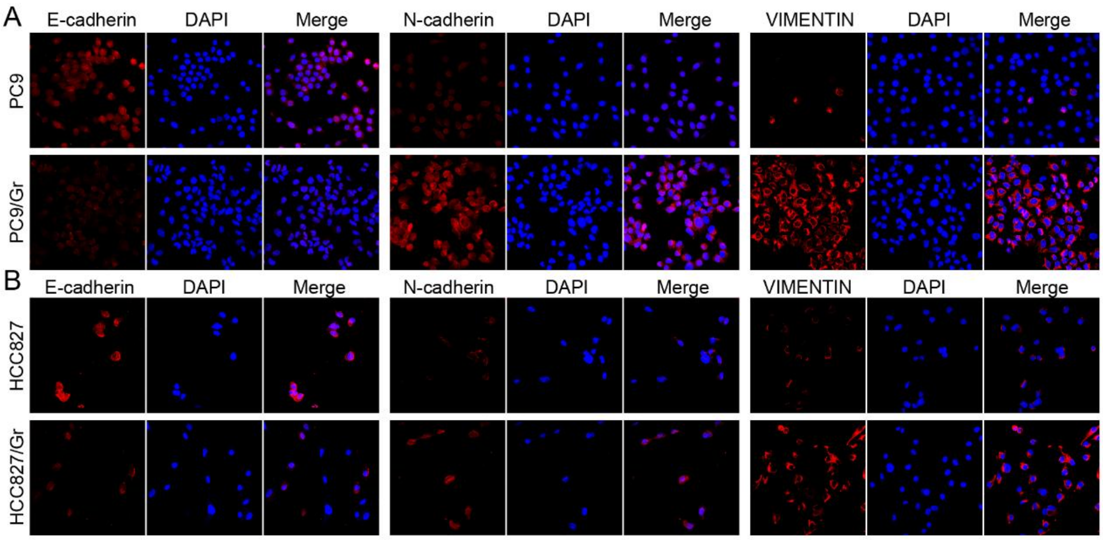

Supplementary Figure S4. A. Immunofluorescence staining of EMT related markers E-cadherin, N-cadherin, and Vimentin in PC9 and PC9/Gr cells. B. Immunofluorescence staining of EMT related markers E-cadherin, N-cadherin, and Vimentin in HCC827 and HCC827/Gr cells.

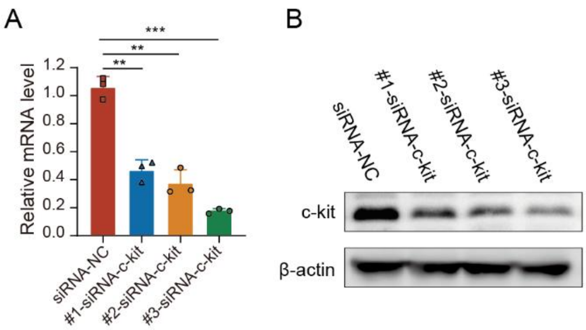

Supplementary Figure S5. A. The relative c-kit mRNA expressions after interference with #1-siRNA-c-kit, #2-siRNA-c-kit, and #3-siRNA-c-kit in PC9/Gr cells were detected by qPCR. B. The relative c-kit protein expressions after interference with #1-siRNA-c-kit, #2-siRNA-c-kit, and #3-siRNA-c-kit in PC9/Gr cells were detected by Western blot. *: $P < 0.05$; **: $P < 0.01$; ***: $P < 0.001$.

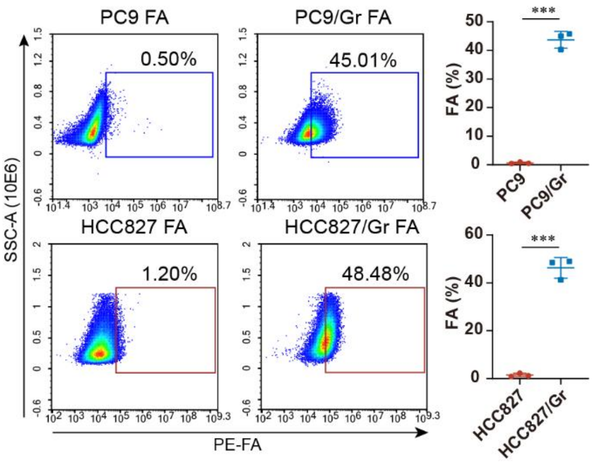

Supplementary Figure S6. Flow cytometry analysis of folate receptor-α in PC9 and PC9/Gr, HCC827 and HCC827/Gr cells. *: $P < 0.05$; **: $P < 0.01$; ***: $P < 0.001$.

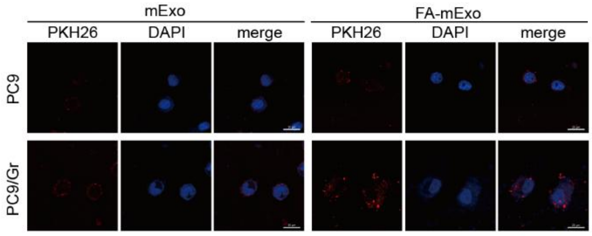

Supplementary Figure S7. Milk exosomes (red fluorescence) with or without FA modification applied on the PC9 and PC9/Gr cells. After 24h incubation, cells were visualized by confocal microscope.

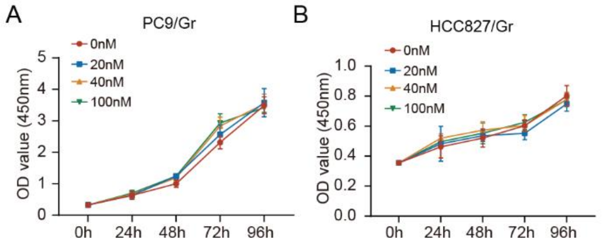

Supplementary Figure S8. Effects of different concentrations of FA on proliferation of PC9/Gr (A) and HCC827/Gr cells (B).

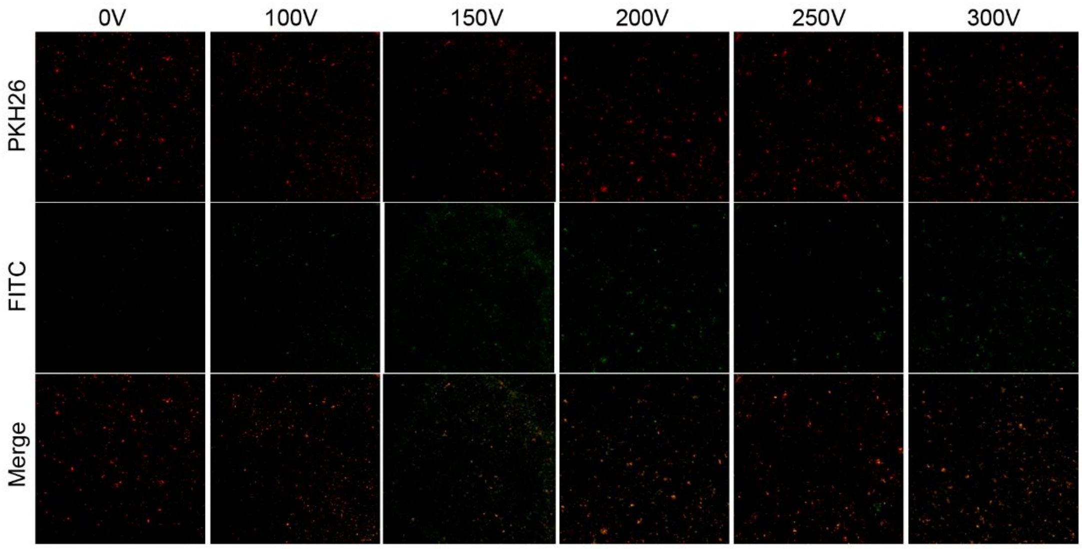

Supplementary Figure S9. The fluorescence intensity of mExo loaded siRNA-c-kit under different electroperforation voltages was observed by confocal microscopy.

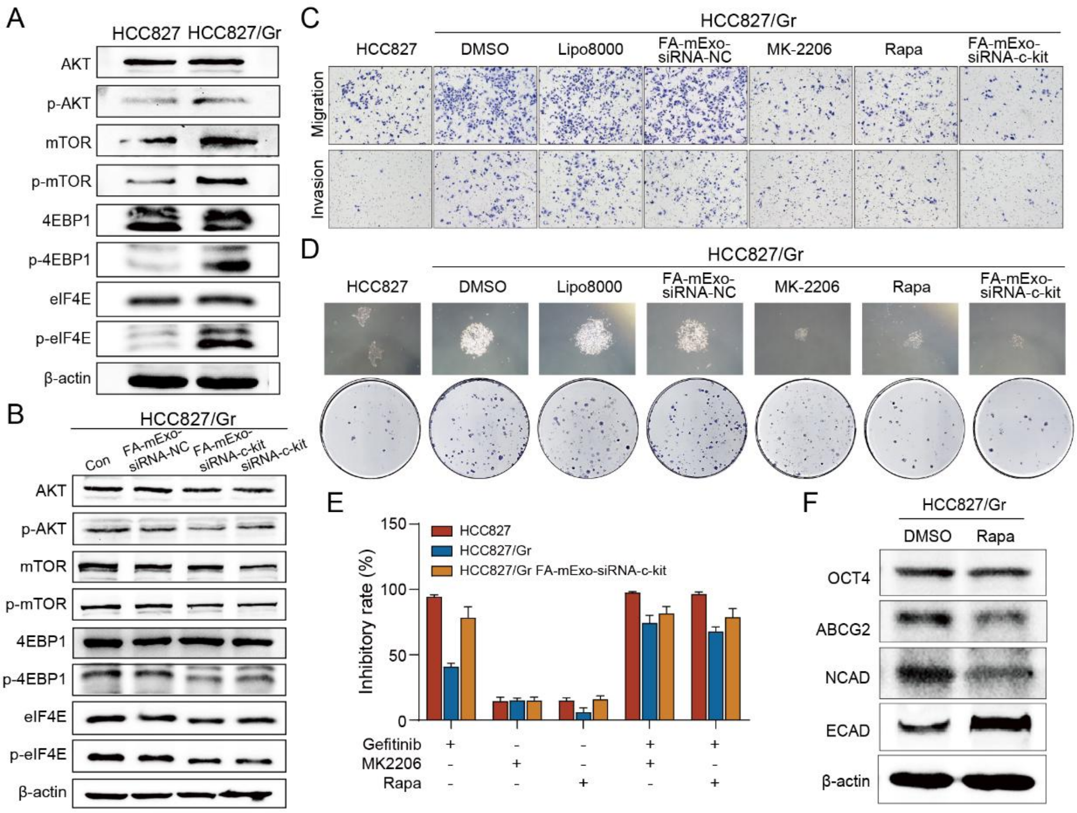

Supplementary Figure S10. A. Protein expression of mTOR signaling pathway-related molecules AKT, p-AKT, mTOR, p-mTOR, 4EBP1, p-4EBP1, eIF4E, p-eIF4E in HCC827 and HCC827/Gr cells. B. Suppression of mTOR signaling pathway after interference of c-kit expression in HCC827/Gr cells. C. The migration and invasive abilities of HCC827/Gr cells were assessed with transwell assays after treatment with MK2206 (AKT inhibitor) or rapamycin (mTOR inhibitor). D. The sphere formation efficiency of HCC827/Gr cells was evaluated after treatment with MK2206 (AKT inhibitor) or rapamycin (mTOR inhibitor). E. Effects of AKT/mTOR inhibitors on gefitinib resistance after blocking the mTOR signaling pathway in HCC827/Gr cells. F. The expression of stem cell-related genes OCT4, ABCG2 and mesenchymal marker N-cadherin was diminished by MK2206 and rapamycin treatment, and epithelial makers E-cadherin expression was increased in HCC827/Gr cells. *: $P < 0.05$; **: $P < 0.01$; ***: $P < 0.001$.

Supplementary Table 1. Primers and probes for qRT-PCR.

KIT_F	CAAGGCTTCTCCAATTCTGC
KIT_R	TGCAGTGGTCCACAGAAGAG
cMET_F	CCAAGTCAGATGTGTGGTCCTTTG
cMET_R	GGTGTTTACGTCAGGATAAGGTGG
HER2_F	GTGTCTGAATTCTCCCGCATGG
HER2_R	CCGCCACTCCTGGTAGATGAG
NANOG_F	CTCCTCCCATCCCTCATAG
NANOG_R	GGCTCCAACCATACTCCA
ABCG2_F	GGGCTTGTGGAAGAATCA
ABCG2_R	GATGGCAAGGGAACAGAA
ALDH1A1_F	TCAAACCAGCAGAGCAAA
ALDH1A1_R	GCCCATAACCAGGAACAA
PROM1_F	GCATCCATCAAGTGAAACC
PROM1_R	ACCAGGCCATCCAAATC
OCT4_F	GTTTGTGGCAGGGCTTT
OCT4_R	TGTGTCCCAGGCTTCTTT
SNAI2_F	CTGCGATGCCCAGTCTA
SNAI2_R	CCCCGTGTGAGTTCTAATG
VIM_1F	GAAGAGAACTTTGCCGTTG
VIM_1R	GAAGGTGACGAGCCATTT
ECAD_F	CGACACCCGATTCAAAGT
ECAD_R	GCGTAGACCAAGAAATGGA
NCAD_F	ATCATTGCCATCCTGCTC
NCAD_R	TCCTCCACCTTCTTCATCA
